# Endocrine and Metabolic Disorders after Hematopoietic Cell Transplantation

**DOI:** 10.4274/tjh.galenos.2019.2019.0248

**Published:** 2020-05-06

**Authors:** Annalisa Paviglianiti

**Affiliations:** 1 Saint Antoine Hospital, Department of Hematology and Cell Therapy, AP-HP, Paris, France

**Keywords:** Diabetes, Metabolic complications, Hematological disease, Hematopoietic cell transplantation, Endocrine disorders

## Abstract

Chemotherapy treatment and autologous and allogeneic cell transplantations are often complicated by the onset of metabolic and endocrine disorders. Autoimmune disorders, metabolic diseases, and hormonal dysfunctions are some of the endocrine complications observed during or after treatment with immunotherapy (mostly novel agents) and/or chemotherapy conditioning for transplantation. Although successful treatment of the underlying hematological condition often improves the dysfunction, endocrinopathies can have an impact on prognosis and are associated with poor survival; therefore, it is important to detect and treat them as early as possible. An increased incidence of cardiovascular diseases and metabolic syndrome has been observed after transplantation mostly in long-term survivors. In addition, chemotherapy and radiation along with the prolonged use of corticosteroids can contribute to the onset of thyroid and gonadal dysfunctions. The aim of this article is to describe metabolic dysfunctions occurring in patients who underwent allogeneic cell transplantation.

## Abstract (tr)

Kemoterapi tedavisi ve otolog ve allojeneik hücre transplantasyonları, metabolik ve endokrin bozuklukların başlaması ile sıklıkla komplike olmaktadır. Otoimmün bozukluklar, metabolik hastalıklar, ve hormonal disfonksiyonlar immünoterapi (çoğunlukla yeni ajanlar) ve/veya transplantasyon için uygulanan hazırlama rejimi sırasında veya sonrasında gözlenen bazı endokrin komplikasyonlardır. Altta yatan hematolojik durumun başarılı tedavisi endokrin disfonksiyonu sıklıkla iyileştirmekle birlikte, endokrinopatilerin prognoz üzerine etkisi olabilir ve kısa yaşam süresi ile ilişkilidir; bu nedenle mümkün olduğu kadar erken saptanmaları ve tedavi edilmeleri önemlidir. Çoğunlukla uzun dönem sağkalan hastalarda transplantasyon sonrası kardiyovasküler hastalıklar ve metabolik sendromun insidansında artma gözlenmektedir. Ek olarak, kortikosteroidlerin uzun süreli kullanımı ile birlikte kemoterapi ve radyoterapi tiroid ve gonadal bozuklukların başlamasına katkıda bulunabilir. Bu yazının amacı allojeneik kök hücre transplantasyonu uygulanan hastalarda metabolik bozuklukların anlatılmasıdır.

## Introduction

Patients with hematological diseases undergoing chemotherapy and/or hematopoietic cell transplantation (HCT) could experience endocrine and metabolic complications affecting their quality of life in a chronic way [1,2,3]. The occurrence of metabolic complications can be related to different factors including hematological disease, preexisting risk conditions, cancer treatments, and HCT conditioning regimen modalities (total body conditioning and type of chemotherapy).

Cancer treatment often consists of a combination of corticosteroids with chemo-immunotherapy that can favor the development of metabolic alterations. Furthermore, the use of immunosuppressive agents in HCT settings is another iatrogenic cause (Table 1). Nevertheless, the majority of available data on the occurrence of endocrine complications refers to pediatric populations. Reports on the endocrine consequences of allogeneic transplantation at an adult age are poorer and disparate.

Progress made in the cure of cancer has allowed for an increase in the numbers of survivors of hematological diseases. Therefore, prevention and prompt diagnosis of early and late endocrine and metabolic complications, which impact a patient’s quality of life, are important. Herein, we discuss the main metabolic and endocrine alterations in patients with hematological malignancies undergoing HCT.

## Diabetes

Hyperglycemia is a frequent metabolic alteration in patients with hematological diseases [4]. Glucocorticoids induce hyperglycemia by increasing insulin resistance through post-receptor insulin signaling defects [5]. Different factors can trigger a preexisting condition of insulin resistance or increase insulin requirements in a previously normoglycemic patient. The main cause of hyperglycemia in patients with hematological malignancies is glucocorticoid treatment, which is frequently part of chemotherapy regimens and is also used for the treatment of acute graft-versus-host disease (GVHD) in patients who underwent HCT. Corticosteroids are able to induce apoptosis of lymphocytes [6] and are an essential part of the treatment for lymphoma [7], acute lymphoblastic leukemia [8], and multiple myeloma [9]. Glucocorticoids are also used for the prevention of acute and delayed chemotherapy-induced nausea and vomiting in association with other antiemetic agents with different doses according to grading [10,11,12].

In allogeneic settings, high-dose steroids are used for 1 to 2 weeks and eventually tapered over 8 weeks or more to treat GVHD [13]. The use of calcineurin inhibitors, such as tacrolimus and cyclosporine, is also associated with hyperglycemia due to a direct effect on insulin biosynthesis and release [14], and with islet cell apoptosis after toxic levels [5]. Another possible cause of hyperglycemia in these patients is the administration of total parenteral nutrition (TPN). Several studies have demonstrated higher hyperglycemia rates in HCT recipients treated with TPN compared to those who were not [15].

Hyperglycemia is associated with adverse outcomes in patients undergoing intensive chemotherapy and HCT, such as increased infections [16], incidence of GVHD [17], and mortality [18,19,20]. A survey of 1089 patients who underwent HCT reported higher incidence of type 2 diabetes in allogeneic but not in autologous HCT cases [21]. Moreover, a higher prevalence of metabolic syndrome was reported in 86 patients who underwent allogeneic HCT, highlighting the importance of glycemia monitoring in this setting [22].

Treatment should be differentiated according to preexisting diabetic status. For patients with type 2 diabetes before chemotherapy, insulin substitution therapy is recommended. In a study of patients with hematological malignancies and type 2 diabetes, an increase of insulin therapy to 1.2 UI/kg a day was necessary [23]. In patients with no previous history of diabetes, treatment can be stratified according to mild (<200 mg/dL), moderate (200-300 mg/dL), or severe (>300 mg/dL) hyperglycemia. Intravenous insulin should be reserved for critical cases. For patients undergoing allogeneic HCT, glycosylated hemoglobin (A1c) and lipid assay should be done once a year. This timing should be shortened to 3 or 6 months for patients who received corticosteroids or calcineurin inhibitors [24].

## Metabolic Syndrome

The International Diabetes Foundation has defined metabolic syndrome as the presence of at least three of the following risk factors: abdominal obesity, triglycerides of more than 1.7 mmol/L, HDL cholesterol of less than 1 mmol/L for men and less than 1.3 mmol/L for women, blood pressure of more than 130/85 mmHg, and blood glucose of more than 5.6 mmol/L or treatments for the last three findings. A high incidence of metabolic syndrome has been reported in HCT recipients [24]. One of the causes is the use of corticosteroids. Allogeneic HCT recipients also have a higher incidence of dyslipidemia compared to autologous recipients [25]. The lifestyle and family history in association with treatments (total body irradiation, acute and chronic GVHD, immunosuppressive treatment) have all been associated with an augmented risk of dyslipidemia and, consequently, metabolic syndrome [26]. Although there are no studies showing the incidence of cardiovascular disease after HCT, a few case reports described the onset of coronary artery disease and early heart failure at a median of 7.5 years after HCT in recipients aged 35 years old [27]. In adult long-term survivors it is recommended to perform screening for glycemia and dyslipidemia annually and a blood pressure assessment at every outpatient consultation. Dietary restrictions and treatment with statins for hypercholesterolemia and fibrate for hypertryglicemia should also be considered.

## Hypoglycemia

Hypoglycemia is a rare event but can occur as a consequence of paraneoplastic production of insulin-like factors. The main pathogenic mechanism for hypoglycemia is IGF-2 secretion. Hypoglycemia may also be due to increased glucose consumption by the tumor [28]. Iatrogenic hypoglycemia has also been reported in patients treated with rituximab [29], tyrosine kinase inhibitors [30], and oral purine analogues [31], as well as in those receiving trimethoprim/sulfamethoxazole [32]; all these drugs are commonly used in the allogeneic HCT setting. A suggested mechanism for antibiotic-associated hypoglycemia is the sulfonylurea-like effect [32].

The optimal therapeutic approach to hypoglycemia is to treat the underlying malignancy. Parenteral dextrose has an immediate effect, while oral glucose administration leads to glycemia in 15 to 30 minutes. For recurrent or chronic hypoglycemia, long-term management includes intravenous corticosteroids and glucagon (0.5 to 1 mg, intramuscularly).

## Pituitary Dysfunctions

The prolonged use (more than 3 months) of steroids at a dose of more than 7.5 mg/day can be associated with inhibition of the production of the hypothalamic corticotrophin-releasing hormone, leading to pituitary deficiency in HCT recipients (secondary adrenal insufficiency). Corticotrophin deficiency can also be caused by total body irradiation (TBI) [33]. Blood cortisol levels and ACTH should be tested in all patients with clinical symptoms and/or those who had long courses of steroids treatment.

Replacement therapy with hydrocortisone is recommended until the adrenal axis recovers [34]. Thyrotropin, gonadotropin, and somatotropin deficiencies may also be associated.

## Thyroid Disorders

Hypothyroidism is one of the most common endocrine dysfunctions occurring after HCT [35]. The incidence varies according to conditioning regimen, and it is increased in the case of TBI. In a retrospective study, 248 patients who underwent HCT (related donors, n=150; unrelated donors, n=70; autologous, n=28) were compared to 317 siblings. Multivariate analysis found that chronic GVHD was associated with a higher risk of hypothyroidism together with other endocrine and vascular diseases for related and unrelated survivors compared to siblings [21]. A more recent retrospective study on acute myeloid leukemia patients reported the presence of positive thyroperoxidase antibodies and more than one allogeneic HCT as being the main risk factors for developing clinical hypothyroidism [36]. In addition, several retrospective studies reported prolonged immunosuppressive therapy, HLA B35 of the donor, and female donor to male recipient mismatch as risk factors for hypothyroidism [37]. Patients should be screened every 6 months in the first year after HCT and then once a year for thyroid function (FT4 and TSH). A retrospective study also reported that HCT recipients have a 3.26-fold higher risk of thyroid cancer compared to the general population [38]. For this reason, a thyroid ultrasound exam is recommended every 5 years after HCT after a normal clinical examination or every year after an abnormal thyroid palpation [38].

## Gonadal Dysfunction

Chemotherapy and radiation can cause infertility according to type and dose [39].

For women, the degree of damage is dependent on the chemotherapy agent and the patient’s age. Salooja et al. retrospectively reported pregnancy outcomes after HCT, indicating that patients who received busulfan and cyclophosphamide are at higher risk of ovarian failure, while cases of pregnancy were described for recipients of only cyclophosphamide [40]. On the other hand, radiation leads to sterility. Data derived from human oocyte models treated with radiation have demonstrated that the lethal threshold is 2 Gy [41].

For males, radiation is the main cause of azoospermia. Chemotherapy with busulfan induces azoospermia at a lower rate (approximately 50% of male patients). Rovo et al. retrospectively reported that the presence of GVHD was associated with adverse sperm recovery in 224 male patients who underwent HCT [42].

## Osteoporosis

The use of corticosteroids for hematological diseases and for acute GVHD treatment is one of the main risk factors for osteoporosis in HCT recipients. Age, lack of physical activity and sun exposure, and gonadal failure are other causes. Moreover, high-dose chemotherapy has been associated with a loss in trabecular and cortical bone [43]. Vitamin D supplementation (100,000 unit dose of oral cholecalciferol every month) is essential to prevent osteoporosis in HCT recipients.

## Conclusion

Survival after chemotherapy and HCT has improved with time; therefore, it is important to include evaluation of metabolic and endocrine disorders during follow-up. Moreover, the impact of haploidentical HCT and novel immunotherapies on long-term outcomes is still under assessment. Prospective research is needed to better define individual risk factors for prevention and strategies for treatment.

## Figures and Tables

**Table 1:**
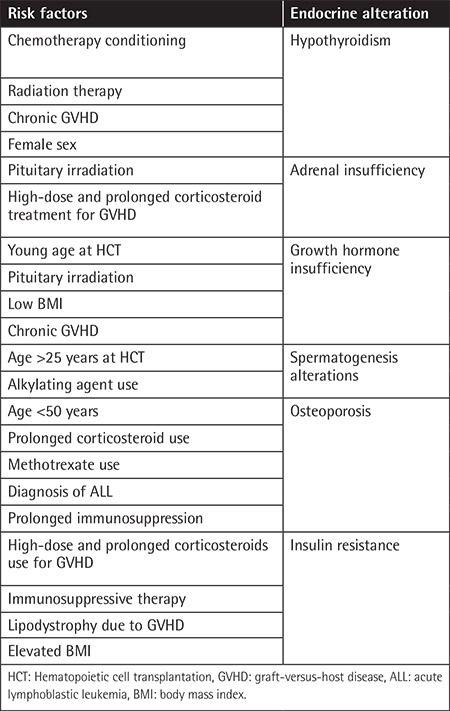
Main risk factors for endocrine disorders after HCT.
